# The role of traditional ecological knowledge, given the transformation of pastoralism in Central and Eastern Mongolia

**DOI:** 10.1007/s13280-024-02057-w

**Published:** 2024-08-29

**Authors:** Sophie Peter, Sarah Niess, Batbuyan Batjav, Nandintsetseg Dejid, Lukas Drees, Yun Jäschke, Ulan Kasymov, Sugar Damdindorj, Khishigdorj Dorjoo, Ganzorig Gonchigsumlaa, Denise Margaret S. Matias, Thomas Müller, Marion Mehring

**Affiliations:** 1 ISOE - Institute for Social-Ecological Research, Hamburger Allee 45, 60486 Frankfurt am Main, Germany; 2 Senckenberg Biodiversity and Climate Research Centre, Georg-Voigt-Straße 14-16, 60325 Frankfurt am Main, Germany; 3 CNPS - Centre for Nomadic Pastoralism Studies, Ard Ayush Ave. 33-18, 4th Sub District, Bayangol District, Ulaanbaatar, 160063 Mongolia; 4 Department of Botany, Senckenberg Museum of Natural History Görlitz, Am Museum 1, 02826 Görlitz, Germany; 5 TUD - Dresden University of Technology, Markt 23, 02763 Zittau, Germany; 6 School of Economics and Business, Department of Agricultural and Applied Economics, MULS - Mongolian University of Life Sciences, “Khan-Uul” District, Zaisan, Ulaanbaatar, 17024 Mongolia; 7 Institute of Geography and Geo Ecology, 2d Building of MAC, Baruum Selbe Street 15-4, 4th Sub District, Chilgeltei District, Ulaanbaatar, 15170 Mongolia; 8 HNEE - Eberswalde University for Sustainable Development, Schicklerstr. 5, 16225 Eberswalde, Germany

**Keywords:** Central and Eastern Mongolia, Inter- and intragenerational knowledge transfer, Pastoralism, Social science mixed methods approach, Traditional ecological knowledge (TEK)

## Abstract

Mongolian nomadic herders traditionally pass on ecological knowledge intergenerationally, mainly within families. However, little is known about how current societal transformation processes may impact the application and transfer of traditional ecological knowledge (TEK) amongst herders. Combining quantitative household survey data with qualitative interviews, we show that TEK is still widely applied amongst herders. Our data show that households living under conditions of greater societal transformation apply TEK more often in order to adapt to the situation than households under lower transformation pressure. High transformation pressure goes along with high human population and livestock density and thus competition for good pastureland. In addition, our results show that intragenerational knowledge transfer between families is gaining more importance nowadays. For Mongolia, we recommend facilitating access to and strengthening the exchange of TEK to prepare herders for the future due to the high level of uncertainty accompanying societal transformations.

**Supplementary Information:**

The online version contains supplementary material available at 10.1007/s13280-024-02057-w.

## Introduction

For centuries, pastoralism in Mongolia has been nomadic, meaning a herder household (HH) or an individual 'who moves frequently and irregularly in a smooth space, and whose mobility is primarily dedicated by environmental factors such as the need for water and pasture and/or climatic conditions' (Myadar 2021, p. 21). The nomadic lifestyle enables sustainable use of the grassland (Sakai and Umetsu 2014; Gantuya et al. 2021) and gives the ability to cope with the extreme weather conditions (e.g. cold blasts known as ‘*dzud*’, with temperatures as low as minus 50 degrees Celcius) in order to protect livestock. A deep and close connection with nature is part of this lifestyle, and the values, attitudes and worldviews of herders (Marin 2008; Drobyshev and Syrtypova 2016). Within this social–ecological system, a knowledge of the ecological conditions, geomorphology and hydroclimate is a key element of the traditional nomadic way of life and is influenced by cultural as well as social and political norms and values (Allegretti et al. 2015; Yembuu 2016). These social structures contain both formal and informal regulations, e.g. for managing common grazing land to prevent its degradation from overexploitation and strengthen its resilience (Jamsran 2010). In Mongolia, the formal regulations changed radically with the political transition from socialism to a democratic system by the early 1990s. Between 1921 and 1991, the country underwent major state intervention, aimed at modernization and industrialization. Collectivization, known as *Negdel* in Mongolian, was one instrument that had a huge impact on the nomadic lifestyle. The state provided infrastructure and basic resources (Batkhishig 2009), and by 1959, 99% of all herder households (HHs) were members of a herding collective (Barzagur 2002; Fernández-Giménez 2006). During this period, scientific knowledge provided by external experts to herders became more important and as a consequence a part of the traditional lifestyle (Jamsran 2010). As a result, a loss of traditional ecological knowledge (TEK) is alleged to have occurred (Fernández-Giménez 2000; Soma and Schlecht 2018).

### Traditional ecological knowledge as a key element of the nomadic lifestyle

Traditional ecological knowledge (TEK), especially in relation to conservation of the environment, has been studied in Western science since the 1980s (e.g. IUCN´s *Programme on Traditional knowledge for Conservation* (1986), *World Commission on Environment and Development* (1987)) (Chapman 2007). As the case of Mongolia shows, the equal and complementary treatment of Western science and TEK is of crucial importance (Knudtson and Suzuki in Chapman 2007). Consequently, the *Intergovernmental Science-Policy Platform on Biodiversity and Ecosystem Services* (IPBES 2022) emphasizes the importance of integrating Indigenous and local knowledge into their work but also into management strategies. TEK is considered to be a knowledge–practice–belief system (Berkes et al. 2000). It is holistic, intuitive and local-specific knowledge about nature conditions in the long term (Fernández-Giménez 2000; Chapman 2007; Diemont and Martin 2009). It reflects a strong symbiotic relationship between humans and nature (Berkes 2010). TEK can be modified by new experiences and is thus flexible and an ongoing learning process (Fernández-Giménez 2000; Pierotti and Wildcat 2000; Chapman 2007; Martin et al. 2010). TEK is applied throughout the world in communities living in highly coupled social–ecological systems (Martin et al. 2010). Most of the research to date has focused on its definition, role, but also its application and potential loss. Especially the latter development is evident on a global scale, mainly due to social, economic, political and ecological drivers (Tang and Gavin 2016; Rai and Mishra 2022; Abdullah and Khan 2023; Singleton et al. 2023). The majority of scientists consider the preservation of this form of knowledge to be essential in order to enable a sustainable transformation and resource management (Pierotti and Wildcat 2000; Menzies and Butler 2006; Armstrong et al. 2007; Rupprecht et al. 2020). However, there are also some critical voices that describe and critically question the application of TEK keeping the people in a poverty trap (Hartel et al. 2023). Consequently, little is known about the role and relevance of TEK under ongoing societal transformation processes. In particular, the influence of urbanization, economic development and the renunciation from traditional environmental management practices on the application of TEK is not well studied. In this context, the overall question is how to integrate TEK into modern structures of practical knowledge. Finally, research lacks an unbiased exploration of the role of TEK as part of the sustainable development of a social–ecological system like Hartel et al. (2023) and Rai and Mishra (2022) recently claimed. Addressing these gaps necessitates a more comprehensive, interdisciplinary and collaborative approach in TEK research, a shift from a Western-centric perspective to a more inclusive, global approach and a more nuanced understanding of the diversity of adaptation amongst TEK holders (Baival and Fernández-Giménez 2012; Tang and Gavin 2016; Hill et al. 2020; Rai and Mishra 2022; Abdullah and Khan 2023; Hartel et al. 2023; Singleton et al. 2023).

In Mongolia, the respectful and caring treatment of nature is part of the pastoralism and TEK plays a prominent role (Purevdorj and Buyanjargal 2019). Traditionally, the way of life of herders is based on practices and routines that are closely linked to the social–ecological system, which ensures survival under the harsh conditions and shapes the ecological system (Gantuya et al. 2021). Herding practices and TEK are highly connected, for example, the knowledge how to deal with extreme events, to assess pasture quality or to treat livestock (Gantuya et al. 2021). As a result, this has lead 'to a flexible, mobile system of pastoral land use that persisted over centuries' (Fernández-Giménez 2000, p. 1324). As already mentioned, the degree to which herders apply TEK has been negatively affected by the socialist era and its top-down approach, and by restructuring after its dissolution (Fernández-Giménez 2000; Soma and Schlecht 2018). However, scientists like Ahearn (2019) point out that TEK plays a prominent role in identifying impacts of climate change and passing this knowledge to other generations. Nevertheless, an empirical study of this loss in Eastern Mongolia is missing.

Like other traditional Mongolian behaviours, TEK is transferred intergenerationally, mostly informal and orally (Yembuu 2016; Singleton et al. 2023). On a global scale, this transfer is often disrupted by societal factors like war and military actions, loss of land tenure, population decline or a shifting lifestyle and education (Haq et al. 2023; Tang and Gavin 2016). Specifically, Rai and Mishra (2022) identified barriers on a practical level, such as  inadequate acknowledgements or records. Thus, the preservation of traditional knowledge resulting from a fluent transfer is recognized in international scientific literature and is attempted through capacity-building and community-based activities (Tang and Gavin 2016, Hill et al. 2020; Fernández-Llamazares et al. 2021). In Mongolia, the process of TEK transfer has also experienced significant social alterations over the past few decades. The nuclear family has always been a central system of knowledge transfer (Soma and Schlecht 2018; Reid-Shaw et al. 2021), but the state has ceased to be a source of knowledge since the collapse of the socialist block under the rule of the former Soviet Union, along with state-organized meetings, books or governmental brochures (Fernández-Giménez 2001). A loss of TEK knowledge, or lack of transfer, could consequently lead to long term and large-scale ecological consequences for the Mongolian steppe ecosystem (e.g. through land degradation because of inappropriate (over)-use) (Fernández-Giménez 2000). To depict this development, empirical studies are rare.

### The transformation of herding across Central and Eastern Mongolia

Since the dissolution of the socialist block, the reality for many Mongolian herders has changed drastically (Yembuu 2016). Enkhbayar (2017) provides a summary of the main consequences of these developments: (1) the ageing of herders; (2) the lack of access to social state benefits for herders; (3) the Mongolian education system, which is not geared to nomadism; and (4) environmental hazards that make it difficult to keep livestock. These developments in turn have an influence on the socio-demographic characteristics of a HH (i.e. age, gender, number of household members, education), but also on their herding patterns (i.e. their experience in pastoralism, traditional seasonal mobility, membership in a pasture user group (PUG) to manage natural resources collectively at local level (Fernández-Giménez et al. 2015), number of livestock). Economic diversification and privatization have replaced strict state regulations and allowed the free market to dominate. However, the government is still setting grazing boundaries, as are the HHs themselves, based on norms, knowledge and perceptions (World Bank 2009; Meurs et al. 2017). Such a process of individualization contrasts with long-term management geared to the common good (Olson in Jamsran 2010). It is accompanied by social consequences such as a worsening of economic inequalities (Fernández-Giménez 2006), and the overshooting of Mongolia’s ecological and material footprints due to changes of land use and nitrogen flows (Fanning et al. 2022). As a result, Mongolian herders face two development dynamics. On the one side, the societal and economic dynamics push them to optimize and increase the efficiency of their herding, which may require them to reconsider their traditional seasonal mobility (Gonchigsumlaa and Damdindorj 2021). On the other side, Mongolian society does not want to lose the nomadic lifestyle and TEK as part of its national identity (Marin 2008; Myadar 2021).

As a result of the societal transformation process, such as urbanization and economic development (Enkhbayar 2017), the spatial distribution of HHs has changed over the last decades (Jamsran 2010, Fraser 2021). Data from the National Statistical Office (NSO) of Mongolia show migration from rural areas such as the eastern region close to the Chinese border over the past 20 years, mainly to central Mongolia but also abroad, which 'impacted traditional land use practices and the local knowledge associated with these practices' (Mehring et al. 2018, p. 4). In 2021, HH density was highest in the central province (or ‘*aimag*’ in Mongolian) of ‘Tuv’ (0.23 HHs per km^2^) close to Ulaanbaatar and considerably lower in the eastern *aimags* ‘Khentii’ (0.15 HHs per km^2^), ‘Sukhbaatar’ (0.14 HHs per km^2^) and ‘Dornod’ (0.07 HHs per km^2^). In Mongolia (2022), 26.9% of herders are aged between 15 and 34, but the majority (73.1%) are aged 35–65 and above (NSO 2023b). Male herders predominate by 59.6% (NSO 2023b). Considering the education level in 2022, the majority has a primary to secondary education (85%). Finally, the number of livestock has risen sharply since the 1970s and even more steeply near the capital compared to the eastern steppe (NSO 2023a). In the long term, these trends of socio-demographic dynamics and urbanization (Drees et al. 2022) might have ecological consequences such as overgrazing or degradation of pasture land closer to the capital (Batkhishig 2013; Yan et al. 2023). In addition, herders are experiencing a degradation and shift of the social–ecological system and thus a weakening of the cultural–ecosystem bond, like the potential loss of the traditional nomadic lifestyle, as a possible consequence (Fernández-Giménez et al. 2016).

**Fig. 1:**
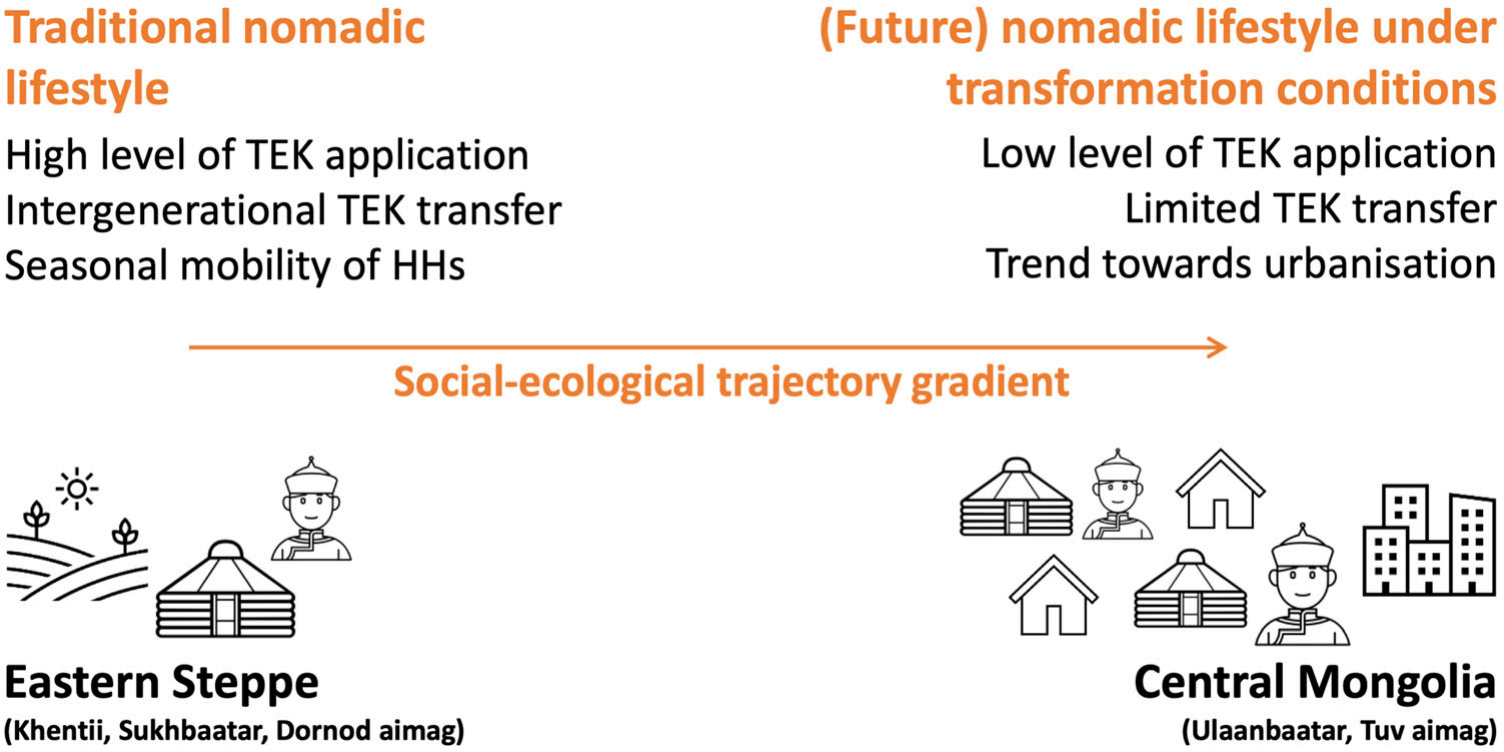
Illustration of the ‘space-for-time substitution’ by the ‘social–ecological trajectory gradient’. The gradient reflects transformation processes over time by visualizing spatial differences between central Mongolia and the Eastern Steppe. It enables to derive assumptions about temporal developments of TEK application, TEK transfer and seasonal mobility of HHs based on the observed spatial differences. (Source: Own illustration; illustrations from the Nounproject by: Agnes Bonmati (Ger tent), Amethyst Studio (Mongolian herder))

Against the background of these transformation processes within the tight social–ecological system of Mongolian nomadism, a ‘social–ecological trajectory gradient’ can be aligned. This gradient stretches from the capital Ulaanbaatar (Central Mongolia) to Eastern Mongolia, ranging from the more densely populated and overgrazed areas around Ulaanbaatar to the less populated and pristine areas further to the east (Fig. 1). Consequently, in Central Mongolia, the competition of herders for good pastureland is high compared to Eastern Mongolia. Thus, herders maintain their livestock under extreme conditions such as grazing intensity and degradation. It is likely that these conditions will intensify under future climate change. In summary, this gradient is a tool for the application of the ‘space-for-time’ substitution (Blois et al. 2013), as it allows the measurement of transformation processes of the nomadic lifestyle in Mongolia between urban spaces and traditional nomadic life in Eastern Mongolia even though temporal changes cannot be measured directly. Thus, the gradient serves as a proxy for temporal trends, and assumptions about changes over time can be inferred from the spatial differences that the gradient represents.

With this article, we seek to better understand the role and relevance of TEK for herders’ nomadic lifestyle in Central and Eastern Mongolia in the light of the ongoing societal transformation. The empirical study thus aims to contribute to a more nuanced understanding of pastoralists’ adaptation strategies in the process of social transformation, also in an international research context. In particular, we test the following hypotheses:

1. Changes in the traditional nomadic lifestyle lead to an erosion of traditional ecological knowledge.
2. Intergenerational transfer of TEK is still the main way of sharing knowledge amongst herders.

With this focus, we contribute to the international scientific discourse on the application and transfer of TEK using empirical, large-scale data from a country under social–ecological transformation such as Mongolia.

### Study area

**Fig. 2:**
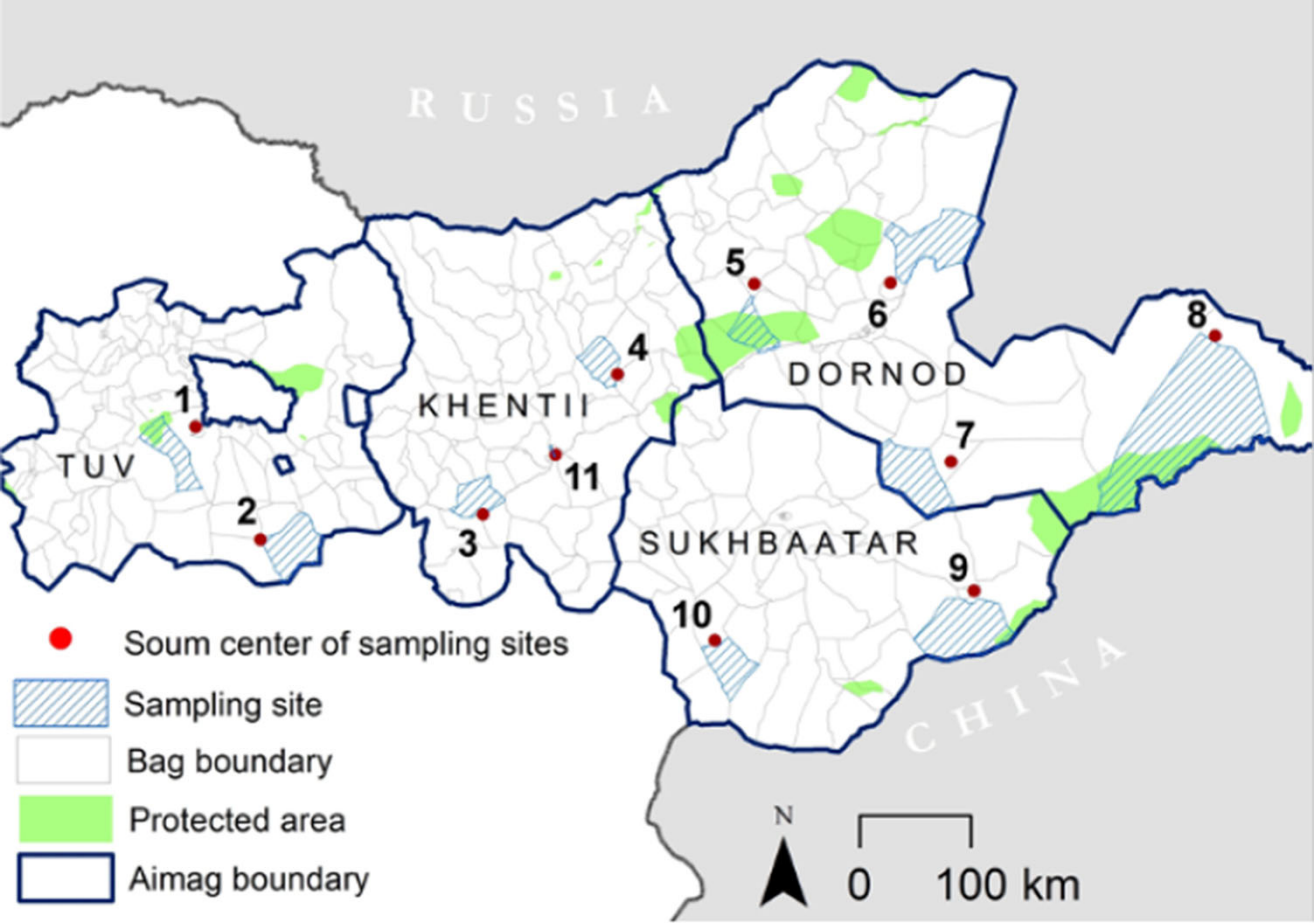
Study area incl. the eleven sampling sites (shaded light blue). Blue outlines indicate the aimag boundaries of Tuv (to the west of the study area), Khentii (directly east of it), Sukhbaatar (to the south) and Dornod (to the east). Protected areas are shaded green; *bag* (rural sub-district) boundaries in grey (Source: N. Dejid, MORE STEP)

The study area covers an area of 369,638 km^2^ within the *aimags* of Tuv, Khentii, Sukhbaatar and Dornod in Central and Eastern Mongolia. For the project, eleven sampling sites were selected for data collection (Fig. 2). These sites represent the Eastern Mongolian Steppe ecosystem, which is the largest mostly intact steppe ecosystem worldwide, characterized by a close coupling of societal and ecological processes (Batsaikhan et al. 2014; Wesche et al. 2016). Thus, the study area provides a unique opportunity to investigate the effects of societal transformation, including changing nomadic lifestyles that in turn affect the Mongolian rangeland and the mobility of herders.

## Materials and methods

The data were collected between 2019 and 2022, except for the year 2021 due to the COVID-19 pandemic. The methodology includes a mixed methods approach: A comprehensive quantitative HH survey was designed to address the first hypothesis on the degree to which TEK is applied, and its sources. In this context, the social–ecological trajectory gradient intents to reflect on the current and future transformation processes of the nomadic lifestyle. Furthermore, we applied a qualitative approach to explore the second hypothesis about the diverse TEK transfer and content, with face-to-face interviews, focus group discussions and in-depth expert interviews, including a social network analysis. By collecting in-depth data, we obtain information about transformative nomadic lifestyle changes.

### Data collection and analysis

#### Quantitative herder household survey

The project team, consisting of German and Mongolian researchers, jointly implemented a HH survey of randomly selected HHs in 2019, 2020 and 2022 using a panel design. In total, 253 HHs along the ‘social–ecological trajectory’ gradient were included in this analysis and repeatedly surveyed in all three years. The HHs surveyed are representatively distributed amongst the eleven sampling sites of the project (see Fig. 2), whereby 53 HHs were surveyed in Central Mongolia (Tuv *aimag*) and 200 HHs in Eastern Mongolia (Khentii, Dornod and Sukhbaatar *aimags*).

In general, the survey has a broad thematic focus on HHs in the eastern steppe. For this publication, the questions on TEK are analysed and presented. We asked seven questions with pre-formulated response options to calculate the ‘degree of TEK application’ as a knowledge basis of their daily herding routines: the selection of pasture quality (e.g. most important criteria for selecting pasture or camp; see Sections E, Q in Appendix Table S1), prediction of and adaptation to extreme events (*dzud* and drought) (Section G), and treatment of sick and injured livestock (e.g. if the animals are sick with disease, how do you treat them?; Section M). Besides that, we use data on the HH structure and livestock, pastureland conditions and utilization, herder mobility, and coping with disasters and livestock loss (see all related variables in Appendix Table S1). The following variables were included in the analysis: socio-demographic information for exploring which factors are influential for the application degree of TEK (i.e. average age; education of all HH members with herding experience; membership of a herder organization); the social–ecological trajectory gradient to obtain data on the effects of social transformation processes (i.e. distance of the HH location to the capital Ulaanbaatar in 2020); the movement frequency of HHs between seasons as a characteristic of a traditional nomadic lifestyle (Fernández-Giménez and Le Febre 2006); and livestock number (i.e. total livestock of the HH transferred in sheep units (SU)). As ‘degree of TEK application’ was collected in the 2020 survey, corresponding household information (socio-demographic variables and social–ecological trajectory gradient) was also taken from this annual data collection. Because of the panel design, it was possible to integrate the average of movement frequency and livestock information over three years to provide robustness to outliers over the years and thus a more representative estimate.

Furthermore, the dataset contains information about the source of TEK. This quantitative information supports the qualitative data collected and serves to answer the second hypothesis. Four questions were asked to identify where the respondent learned how to deal with *dzud*, drought, and sick and injured animals. For example, the respondents had six response options to choose from where they learned about the prediction of *dzud* and drought (i.e. parents, other relatives, friends, school, self-taught, training by a government organization or NGO), and the option to formulate their own response (Sections G, M; see Appendix Table S1). The questions relating to the degree of application and source of TEK were asked only once in the 2020 survey like the questions about the TEK application.

All data analyses were done using ‘R’ (R Core Team 2023). The individual analysis steps are required to validate whether the measured constructs are suitable for testing hypothesis 1. As the central construct ‘degree of TEK application’ has not yet been tested or developed in the literature, a factor analysis was conducted as the preliminary step of the analysis in order to validate the measurement scale. Factor analysis is commonly used to identify the shared variance amongst the items and assess the appropriateness of operationalization. In this way, we can assess whether the seven selected questions accurately reflect the ‘degree of TEK application’ (see ‘Data collection and analysis’). We therefore conducted a factor analysis using the ‘fa()’ function from the ‘psych’ package (Revelle 2017). Prior to conducting the factor analysis, several preliminary tests were performed. The Kaiser–Meyer–Olkin (KMO = 0.63) and Bartlett’s test for sphericity (*χ*^2^(21) = 166.26, *p* < 0.001) indicated that the correlations between the items were sufficient to perform a factor analysis. The Velicer’s minimum average partial (MAP) test and visual analysis of the scree plot suggested that the optimal number of factors to retain is 1. Overall, the results of the factor analysis suggest that the items represent the intended construct of ‘degree of TEK application’ well (see Appendix Fig. S1). The ‘degree of TEK application’ is therefore calculated by summing up the responses through a score system to the previously described dimensions on various herding management topics that were answered in accordance with TEK (i.e. if the survey participant answered a question on herding practices with an answer option that is assigned as TEK, the score increased by one point; see Appendix Table S1. Respective variables are marked with ‘TEK’). The maximum possible score for ‘degree of TEK application’ by a HH was 36 points, while the minimum possible score was zero points. Thirty-six points would mean that the household had indicated for all seven questions that it applied the whole range of TEK in their nomadic lifestyle, whereas zero points would mean that the HH did not apply any TEK at all.

As a next step, we employed a two-step analytical approach to explore the bivariate associations between the ‘degree of TEK application’, the social–ecological trajectory gradient, moving frequency between seasons, livestock and socio-demographic variables (see Table 1). First, using the ‘R’ ‘stats’ package (R Core Team 2023) a correlation matrix as an exploratory analysis was conducted, which allowed us to assess the strength and direction of the associations and identify additional explanatory factors for ‘degree of TEK application’ and moving frequency that could capture potential changes in the traditional nomadic lifestyle. The categorical variables in the dataset were transformed as follows to ensure their inclusion in the analysis: membership of a herder organization was dummy-coded, and educational level was included as an ordinal scale (1–6), while gender indicates the proportion of women in a household.

**Table 1:** Means and standard deviations and correlations between the variables ‘degree of TEK application’ (2020 survey), the social–ecological trajectory gradient (SETG: 2020 survey), key socio-demographic HH variables (2020 survey), grazing patterns (average 2019, 2020, 2022 surveys), herding experience in years (2020 survey) and livestock number (average 2019, 2020, 2022 surveys)

	M	SD	Min	Max	1	2	3	4	5	6	7	8	9	10	11
1. Degree of TEK appl	15.5	4.2	3.0	27.0											
2. SETG [km]	413.5	231.3	67.0	921.9	− 0.15										
3. Moving frequency between seasons	3.2	0.9	0.0	4.0	− 0.09	− 0.29									
4. Herder organization	0.6	0.5	0.0	1.0	− 0.16	− 0.08	0.06								
5. Total Livestock [SU]	1169.4	999.6	82.0	6282.9	0.03	− 0.15	0.24	0.17							
6. HH size	4.2	1.6	1.0	9.0	0.03	0.05	0.00	0.16	0.28						
7. Gender [%]	0.5	0.2	0.0	1.0	0.00	0.08	− 0.04	0.05	0.03	− 0.07					
8. Age [yr.]^1^	42.9	11.6	21.7	80	0.23	− 0.19	− 0.07	− 0.04	− 0.06	− 0.38	− 0.06				
9. Education^1^	3.3	0.9	1.0	6.0	0.12	− 0.22	0.09	− 0.04	0.17	− 0.04	− 0.09	− 0.02			
10. Total Income [MNT]	3.047 M	3.456 M	0.00	16.448 M	− 0.03	0.00	− 0.02	0.04	− 0.09	0.11	− 0.10	0.28	− 0.01		
11. Herding experience^1^ [yr.]	20.3	11.1	2.0	63.0	0.13	− 0.11	− 0.08	− 0.01	− 0.01	− 0.29	0.00	0.74	− 0.27	0.19	

For detailed explanations of the variables, see ‘Data collection and analysis’

Spearman correlations; n = 253

^1^average of all HH members that are herders; 1. TEK appl.Degree [0–36]; 2. SETG = social–ecological trajectory gradient, i.e. distance of HH location in 2020 (summer camp; km); 4. Herder organization (e.g. PUG) [0 = No member, 1 = member]; 5. SU = sheep unit conversion of livestock: Camel = 5 sheep, cattle = 6 sheep, horse = 7 sheep, goat = 0.9 sheep; 6. HH size = number of HH members; 7. Gender = % female of HH; 8 = age in years; 9. Education: 1 = No schooling, 2 = Attended primary school, 3 = Secondary school, 4 = High school, 5 = college, 6 = University; 10. Total Income of all HH members; 11. Herding experience = Average years worked as herders

Source: HH survey

Subsequently, using the ‘sem()’ function of the ‘lavaan’ package (Rosseel 2012), a structural equation modelling (SEM) analysis was performed to assess dependency relationships amongst the variables and test our first hypothesis. The SEM model includes three paths to examine the interdependencies between the endogenous variables: the ‘degree of TEK application’, the social–ecological trajectory gradient and the movement frequency between seasons. Additionally, the model incorporates exogenous socio-demographic factors (age, education, membership of herding organization) and the total number of livestock, which was assumed to increase movement frequency of HHs. These variables are connected to the endogenous variables, forming a comprehensive analysis of the relationships within the model. To account for the substantial variation in measurement scales and improve model convergence and stability, all variables were z-standardized prior to analysis.

These results were complemented with an analysis of the source of TEK as part of the analysis of the second hypothesis. The source of TEK was statistically tested by means of four separate questions (see ‘Data collection’). Response options were categorized into ‘family’, ‘community’, ‘formal’ and ‘self-taught’. The frequency of mentions for each category was then analysed descriptively to understand the predominant sources of TEK mentioned by the participants. This analysis provided insights into the relative importance and contribution of different sources in the acquisition of TEK amongst the HHs surveyed.

#### Qualitative interviews and focus groups

In addition to the question of TEK application, we are interested in the transfer and content shifts of TEK to test the second hypothesis. We conducted qualitative interviews with 301 HHs in the years 2019 to 2022 (excl. 2021) at the same 11 sampling sites (see Fig. 2). The interview partners were chosen based on relevance to the topic, following the snowball sampling technique (i.e. interviewees recommend new interviewees). The interview guidelines consisted of six topics, surveyed with 34 open and closed questions: (1) socio-demographic information; (2) HH mobility patterns (i.e. pasture use, changes in pasture use and risk management); (3) general questions on herding, and questions regarding social communities/networks; (4) TEK knowledge sources and sharing, forecast knowledge; (5) pasture conditions; (6) number of livestock (see Appendix Table S3). Questions were selected from the interview guideline based on the socio-demographics of the interviewees. The interviews were gender balanced across the data collection years, and transcribed and analysed by the coding method (Mayring 2014), based on the content of the interview guideline. In parallel, individual expert interviews were conducted with officials such as *soum* (i.e. administrative division) officers, *soum* environmental officers and land officers. The expert interviews concentrated on additional information about the interviewees’ visions of change, with a special focus on policy, perceived ecological changes and their perception of herders. This was important to gain deeper insights into the knowledge and practice sharing of herders. This step was not performed at all sampling sites, as empirical saturation had already set in after 47 interviews (Saunders et al. 2018). The outcome of this step is a diagram of social networks on the transfer of knowledge.

In order to get an even more detailed picture of the answer to the second hypothesis, a total of 24 focus groups were organized across all sampling sites in 2019, 2020 and 2022 based on the interview results. Group discussions, or focus groups, are used to obtain information about a particular topic through everyday conversation and interactions within the group (Biggs et al. 2021). The objective of the focus groups was to target specific herders (e.g. young herders) and gain deep insights into their herding practices (Appendix Table S3). Participants were mostly men. The groups comprised approximately eight respondents. To supplement the qualitative interviews, the discussions were recorded and transcribed, before being coded based on topics from the interview guideline. The results emerge from a joint analysis of the mixed methods approach.

## Results

### TEK continues to be widely applied by herder households

The HHs surveyed consisted of 4.2 members on average, 52% of whom were women, despite 95% of the household heads being male. Overall, 69% of HHs had at least one member who had attended high school or higher education. The mean total income reported was 3047 M Mongolian Tugrik (MNT). The average years of herding experience amongst herders in the households were 45 years. A total of 58% of households were PUG members. In terms of movement frequency between seasons, HHs reported moving between 0 and 4 times per year. On average, they moved 3.2 times annually. The households had an average of 1169.4 sheep units (a common unit in Mongolia for aggregating different animal species, taking into account different forage requirements). The median distance to Ulaanbaatar, representing the ‘social–ecological trajectory gradient’, was 413.5 km.

**Fig. 3:**
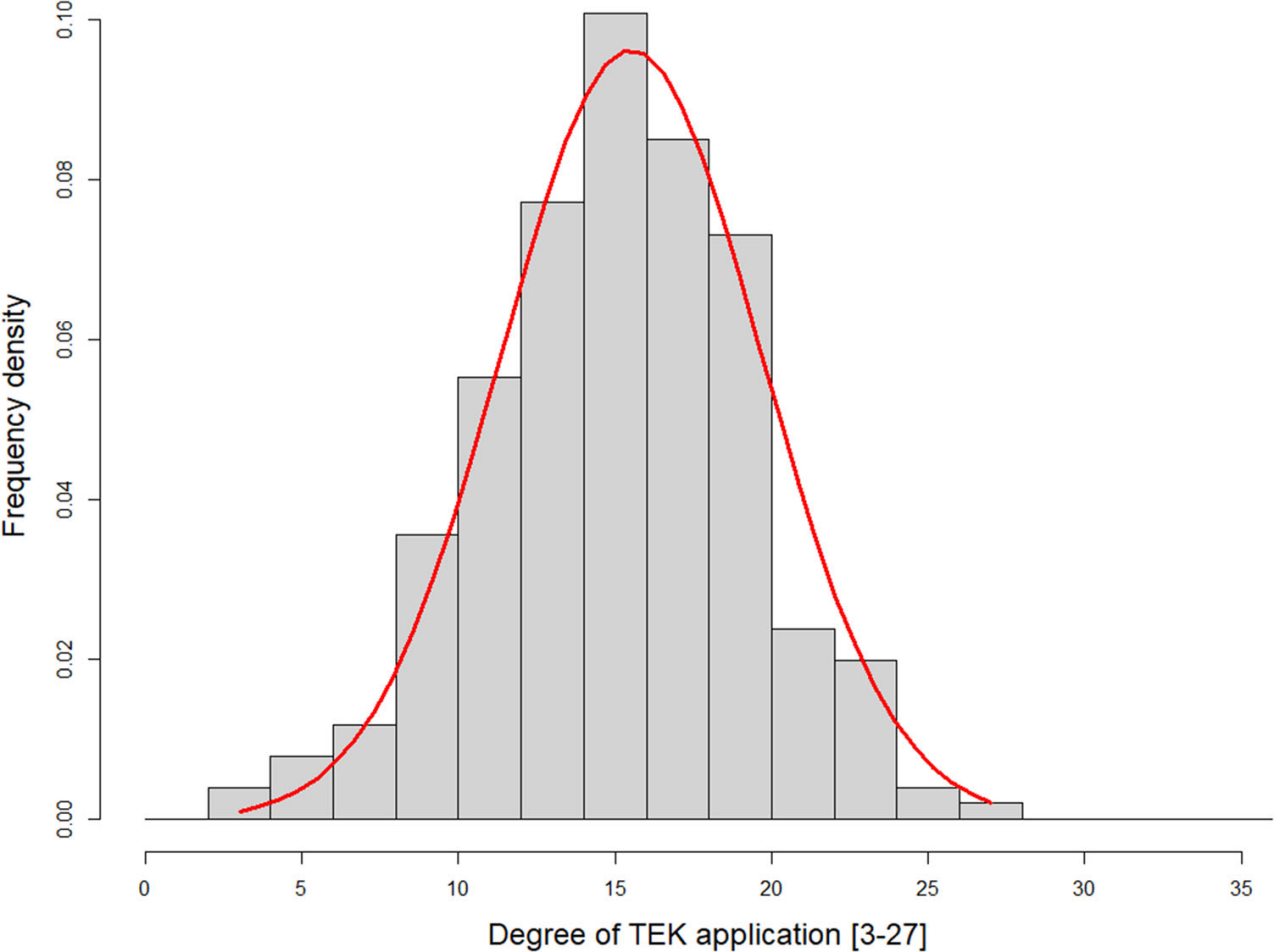
Histogram of degree to which traditional ecological knowledge is applied (*N* = 253). Source: HH survey (2020)

With regard to the first hypothesis, TEK is applied to a strong degree amongst HHs in Central and Eastern Mongolia, with 31% of HHs reaching half the maximum achievable score (18 points or more). The average ‘degree of TEK application’ was 15.5 points, with a standard deviation of 4.2 points (2020, *N* = 253). The results within this sample range from 3 to 27 points and show a normal distribution (Fig. 3), which is also supported by the Shapiro–Wilk normality test (*W* = 0.99, *p*-value = 0.075) (detailed results of the selected seven questions to address ‘degree of TEK application’ in Appendix Table S2).

### Diverse pastoralism practices along the social–ecological trajectory gradient in Central and Eastern Mongolia

In order to investigate the first hypothesis, further variables reflecting the social–ecological trajectory gradient were integrated into the analysis such as the mobility of HHs and other herding-relevant information. Table 1 displays the means, standard deviations, as well as the minimum and maximum values, along with the Spearman correlations between the variables ‘degree of TEK application’, the social–ecological trajectory gradient, key socio-demographic household variables, moving frequency between seasons, herding experience in years and the number of livestock. Results showed that the ‘degree of TEK application’ is negatively related to the social–ecological trajectory gradient (*r* = − 0.15), membership in a herder organization (− 0.16) and the movement frequency of HHs between seasons (*r* = − 0.09), while it is positively related to age (r = 0.23), herding experience (*r* = 0.13) and the highest education level (*r* = 0.12). Along the social–ecological trajectory gradient, we find correlations indicating that the movement frequency of HHs between seasons is negatively related to the social–ecological trajectory gradient (− 0.29). Also age (*r* = − 0.19) and education (*r* = − 0.22) are negatively correlated with the social–ecological trajectory gradient. Gender, income and HH size do not show strong correlations with the ‘degree of TEK application’ and are thus excluded from further SEM analysis. Herding experience is strongly correlated with age (*r* = 0.74), as it is conceptualized and measured as the average number of years HH members have worked as herders. Therefore, only age is considered in the next step of structural equation modelling.

### Application of TEK higher under transformation conditions compared to traditional rural areas

**Fig. 4:**
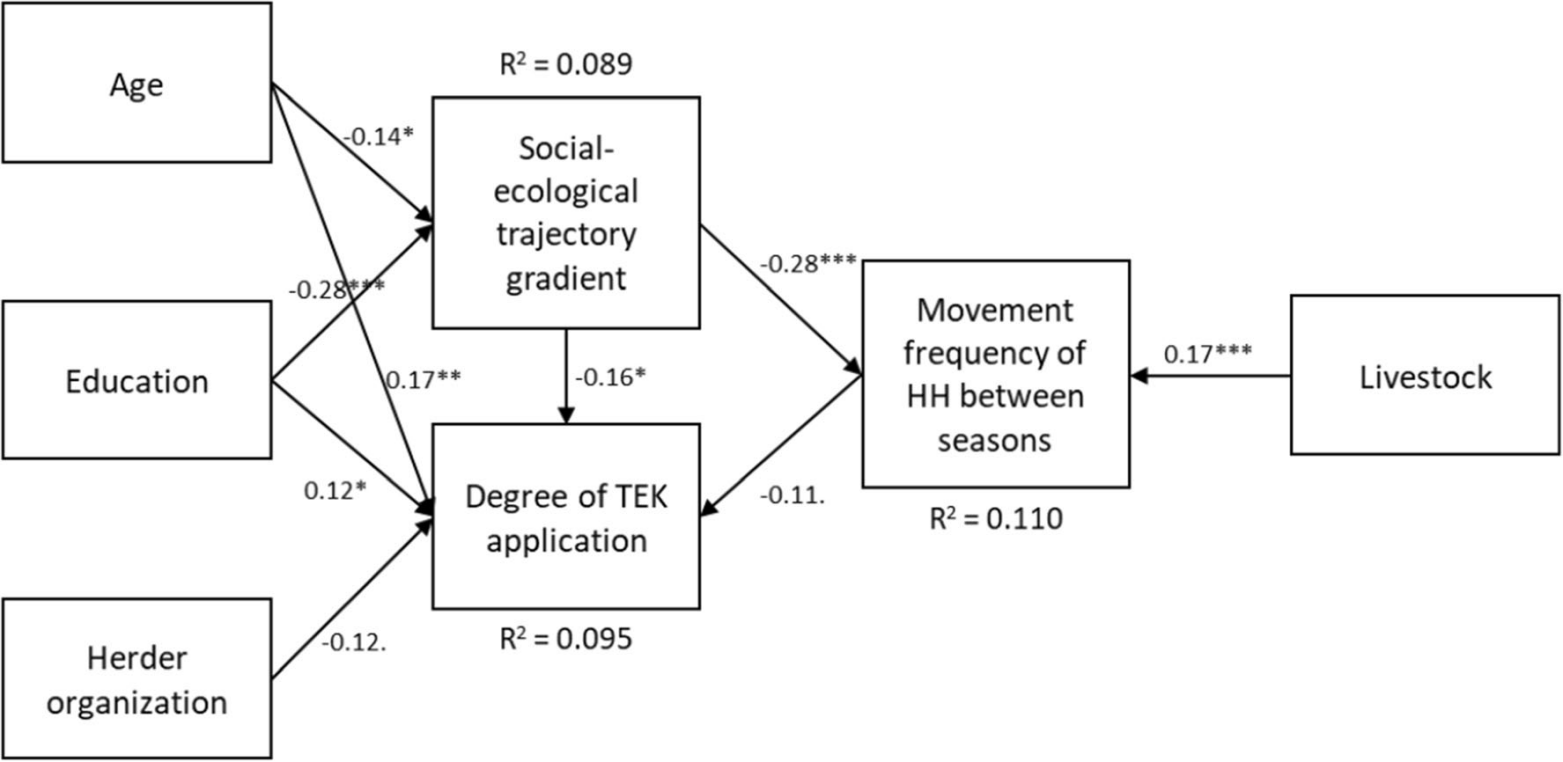
Structural equation model for the associations of ‘degree of TEK application’ (Source: 2020 survey) and movement frequency of HHs between seasons (source: average 2019, 2020, 2022 surveys) along the social–ecological trajectory gradient and with age, education and membership in a herding organization (Sources: 2020 survey) as well as number of livestock (Source: average 2019, 2020, 2022 HH surveys). ***p < 0.001; **p < 0.01; * < 0.05;  < 0.1; Notes: 1. Coefficients shown are standardized paths. To enhance visual clarity, loadings of covariants and error terms are not shown

A structural equation model was applied to analyse the complex relationships between the relevant variables as part of the first hypothesis. SEM analysis included three paths to investigate the relationships between the variables that constitute the first hypothesis (moving frequency and ‘degree of TEK application’ along the gradient), plus additional exogenous factors (Fig. 4). Firstly, the path from ‘age’ and ‘education’ to ‘social–ecological trajectory gradient’ revealed a significant negative relation, indicating that a greater distance to Ulaanbaatar was associated with a lower average age (*β* = − 0.14, *p* = 0.025) and a lower level of education (*β* = − 0.28, *p* < 0.001) (*R*^2^ = 0.089). Secondly, the path from the ‘social–ecological trajectory gradient’ to ‘degree of TEK application’ (*β* = -0.16, *p* = 0.016) demonstrated a significant negative relation and ‘membership in a herder organization’ (*β* = − 0.12, *p* = 0.063) as well as ‘moving frequency’ (*β* = − 0.11, *p* = 0.095) showed a marginally negative relation. ‘Age’ (*β* = 0.170, *p* = 0.008) and ‘education’ (*β* = 0.12, *p* = 0.044) are positively related to the ‘degree of TEK application’, suggesting that a higher ‘degree of TEK application’ was associated with a shorter distance from Ulaanbaatar, no membership in a herder organization, lower movement frequency, a higher age and a higher education level (*R*^2^ = 0.095). This result contradicts the first hypothesis that the further east in the steppe the HHs are located, the higher the ‘degree of TEK application’. In fact, it suggests a reverse effect: the closer the HHs are to Ulaanbaatar (meaning that they presumably lead a nomadic way of life under transformation conditions), the higher the ‘degree of TEK application’. Thirdly, the path to the endogenous variable ‘moving frequency’ reveals a significant negative relation along the gradient (*β* = − 0.28, *p* < 0.001), while more livestock leads to higher moving frequency (*β* = 0.17, *p* = 0.006) (*R*^2^ = 0.11). The third path also contradicts the first assumption and reveals a reverse relation along the gradient: the further east in the steppe the HHs are located, the lower the moving frequency between seasons, which is also an indication of nomadic way of life under transformation conditions (Fig. 4).

Multiple fit indices were utilized to assess the overall fit of the model and the fit of specific paths or relationships within the model. The Chi-square test of model fit indicated that the model fitted the data well (*χ*^2^(6, *N* = 253) = 5.71, *p* = 0.46). Additionally, the Root Mean Square Error of Approximation (RMSEA) was found to be 0, also indicating a good fit. The same was true for the Comparative Fit Index (CFI), which yielded a value of 1. These findings collectively support the notion that the model adequately captures the relationships amongst the variables.

### ‘Kinship’ as a primary source of TEK under transformative pressure

In addition to the application of knowledge, we are interested in the question of whether the traditional intergenerational transfer of knowledge continues to dominate or is supplanted by other sources of knowledge (2nd hypothesis). This was mainly investigated using a qualitative methodology, although the quantitative HH survey also included questions about the source of knowledge in addition to the TEK application. The quantitative results show that most HHs indicated their parents as a source for predicting a *dzud* (66%) and drought (66%). The results were similar when respondents were asked about the use of remedies when livestock were sick (66%) or injured (86%). Only 18 HHs did not mention sources categorized as ‘family’ (parents, other relatives, grandparents), while 116 HHs mentioned ‘family’ as a single source. ‘Self-taught’ is the second most mentioned source of information. A combination of information sources from ‘family’ and ‘new sources’ (i.e. friends, school, self-taught, training by governmental organizations, NGO) was mentioned most frequently by 119 HHs. Furthermore, when regressing the mentioning of ‘family’ as a source of TEK in the four categories (i.e. *dzud*, drought, sick and injured animals) against the ‘degree of TEK application’, for each additional time HHs report family as a source, the ‘degree of TEK application’ increases by 0.946 (*R*^2^ = 0.22, *F*(1, 251) = 73.53, *p* < 0.001).

**Fig. 5:**
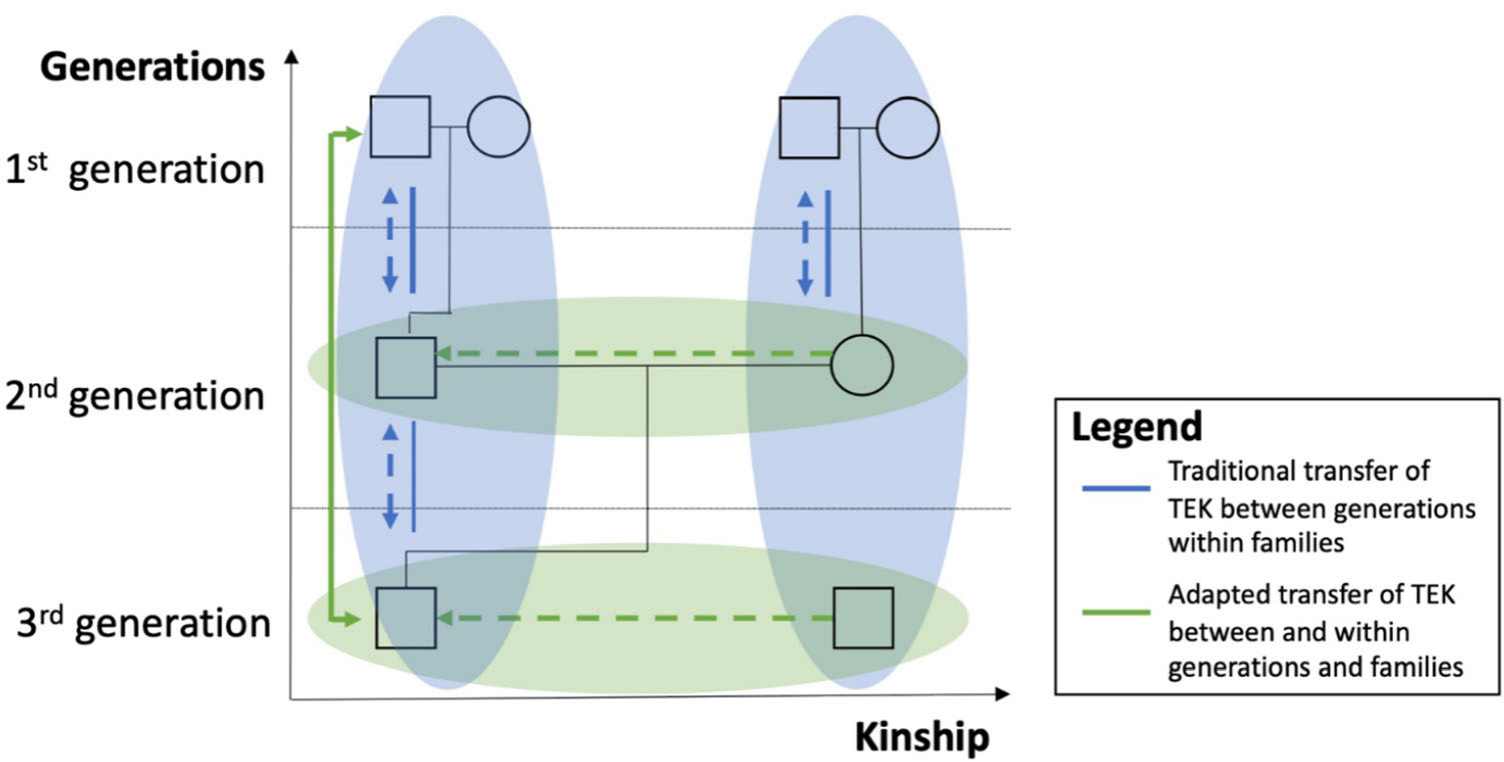
Inter- and intragenerational transfer of TEK across generations within families. The rectangle and circle symbols represent the man and woman of a generation. The solid black lines mark the demarcation between generations. The blue linkages show the local transfer between generations within families. A distinction can be made between top-down (solid lines) and two-way (dashed lines) transfer connections within the family. The green linkages show new and adapted knowledge transfer between and within generations and families: between old and young generations within a family (solid lines); and horizontally within a generation and an expanded kinship and community (dashed lines). (Source: Qualitative interviews and focus groups; own illustration)

The qualitative data collection enabled a more detailed, in-depth analysis of TEK transfer within and between families. The results show that traditional intergenerational knowledge transfer (vertical intergenerational communication—kinship relation, see Fig. 5) between parents and their children remains an important source of TEK within a family (blue linkages, Fig. 5): '*It is true if in autumn the first snow is mixed with rain, it will snow less…I heard it from my parents…It’s true…this is confirmed by observation over the last two years*' (Sampling Site No. 10 Bayandelger, male, 30 years). When asked directly about the source of knowledge (see Appendix Table S3), the herders interviewed indicated that 50–60% of the knowledge was inherited from their parents. Knowledge transfer between the older generation (60 years and older) and youth (35 years and younger) is important (age categorization is based on the official categorization of the National Statistics Office, solid green linkage, Fig. 5), for example in cases where the parents of the younger generation stopped being herders. In addition to this traditional transfer of knowledge within the family and across its generations (blue ovals), the interviews reveal a new pattern of intragenerational transfer (green oval, Fig. 5). The need for this interaction is increasing due to rapidly changing social and environmental conditions: '*The basic knowledge is inherited from their parents. However, it is only 40–50 per cent of my traditional ecological knowledge…some knowledge is not valid anymore due to changes in the environment, and socio-economic conditions*' (Sampling Site No. 4 Batnorov, male, 35 years). A new aspect in this context is the sharing of the TEK source across nuclear families, or kinship, which leads to diversified, flexible transfer options (*x*-axis, Fig. 5). This gives young herders a chance to learn from other herders and their knowledge when intergenerational knowledge transfer cannot occur, or ecological conditions have changed so drastically that local TEK is no longer applicable (e.g. gazelles are disappearing and can no longer be used as an indication of good forage). A 45-year-old man from Sampling Site No. 4 Batnorov pointed out that '*instead of my father, my traditional ecological knowledge comes from my father-in-law, and even nowadays I learn from him by camping together…also from the broadcast of ‘Malchin TV’ channel*' (blue linkages, Fig. 5). The quotation shows that an ‘intensive’ TEK exchange within the social network of families (horizontal) can be seen (Fig. 5). The importance of horizontal knowledge transfer means the role of social networks is becoming increasingly important compared to the traditional kinship relations.

Furthermore, a main finding of the qualitative mixed methods approach is that different topics are shared in different ways (Table 2). Table 2 indicates a general trend in Mongolia where older generations are more likely to rely on their environmental awareness and pass on this knowledge about ecological conditions. As these conditions are changing due to societal and ecological developments, the results seem to suggest that younger generations are refocusing on possible ways to adapt their own behaviour—for example, by improving livestock breeding, an area that still provides scope for individual intervention and the possibility of higher productivity and profitability.

**Table 2:** TEK sources across generations, their share in knowledge transfer and main topics of TEK transfer

No	TEK source	Share as a source in overall TEK transfer	Topics of TEK transfer
1	Grandparents (1st generation)	Medium	*Ecological conditions* Protection of wildlife Environmental protection Weather forecasting *Nomadic lifestyle* Risk management
2	Parents (2nd generation)	High	*Ecological conditions* Weather forecasting *Nomadic lifestyle* Risk management Otor movement (i.e. long-distance movement) Protection of winter pasture Conservation/ protection of hay meadows Preservation of pastures Selection of seasonal pasture Rotation of grazing areas Daily grazing Improvement in breeding
3	Father-in-law (2nd generation)	Medium	*Ecological conditions* Weather forecast *Nomadic lifestyle* Selection of seasonal pastures Improvement in breeding
4	Uncle (2nd generation)	Medium	*Ecological conditions* Weather forecast Protection of wild animals *Nomadic lifestyle* Risk management Selection of seasonal grazing areas
5	Brother and sister (3rd generation)	Low	*Nomadic lifestyle* Selection of seasonal grazing areas Otor movement
6	Brother-in-law (3rd generation)	Low	*Nomadic lifestyle* Improvement in breeding
7	Friends/ distant relatives/ local herders	Medium	*Ecological conditions* Weather forecasting Protection of wild animals *Nomadic lifestyle* Risk management Selection of seasonal grazing areas Improvement in breeding

Source: Qualitative interviews and focus groups

## Discussion

Our first hypothesis focused on the theme that TEK is relevant and applied as part of the traditional nomadic lifestyle. We were able to show a high degree of TEK application in HHs across the region, with results indicating no general sign of loss. We can thus conclude that TEK is not only preserved (e.g. Fernández-Giménez 2000) but also applied. Based on our analysis, herders in Central and Eastern Mongolia seem to be transferring TEK to their everyday practices, which would suggest its importance, value and prevalence in Mongolian society. This development does not follow the international trend of a loss of traditional ecological knowledge and the resulting call for preservation from the scientific community (Fernández-Llamazares et al. 2021). It rather indicates the high status of TEK amongst Mongolian herders in contrast with other nations (Hartel et al. 2023; Pilgrim et al. 2008). In addition, the structural equation model shows that Mongolian herders under transformation conditions particularly fall back on TEK. This finding is against our initial hypothesis. The results clearly show that the degree of TEK application is higher in areas with high transformation pressure. We interpret this finding as evidence that the degree of TEK application becomes more important the higher the pressure from current societal transformation. As the data indicate, TEK is an important source of knowledge to cope with these circumstances. Our study thus empirically confirms the IPBES’ assessment claiming the need for a better integration of TEK into sustainable development activities (Hill et al. 2020). For example, herders with traditional knowledge on pastureland can better find suitable pasture for their livestock and apply traditional remedies to keep their livestock healthy. In this context, TEK could be classified as secure knowledge that provides certainty in uncertain times.

This high transformation pressure goes along with high human population and livestock density and thus competition for grazing land. As the results, our data show that herders are more mobile (seasonal frequency) closer to Ulaanbaatar compared to the rural areas of the East. Tarne et al. (2022) showed that since the 1970s herders have gradually migrated close to the capital. As a result, experienced, well-educated herders live close to Ulaanbaatar in Central Mongolia, which could influence the decision-making for herding decisions. This is in line with our results and draws attention to education, which, besides the traditional TEK transfer between generations, is an important driver when it comes to applying TEK. In addition, membership in a herder organization, like a pasture user group (PUG), shows no positive but a weak negative correlation with the degree of TEK application indicating that PUGs do not have much relevance for TEK application and conservation. It remains to be seen if PUGs could be a target group to perpetuate TEK and its application amongst herders in the long term.

Looking to the future, the results indicate that the application of TEK might gain even more importance, assuming that the outlook in many parts of the study region will start to resemble the social–ecological trajectories close to Ulaanbaatar. With our quantitative analysis, we can confirm the high relevance of TEK and its application under transformation conditions. Thus, the results show that the application is an important part of the herding practice, especially under pressing conditions, and that the application can therefore adapt to new requirements (Chapman 2007; Fernández-Giménez 2000). In fact, flexibility and adaptability are a key characteristic of TEK, as defined in the scientific literature, in contrast to a static state of knowledge that must be preserved (e.g. Chapman 2007). This highlights the close coupling between ecological conditions (e.g. condition of pasture land) and the application of TEK, and thus the high potential to foster resilience (Ruiz-Mallén and Corbera 2013). The results show that the application of TEK enables herders to adapt to new conditions. Additionally, the qualitative data provide valuable evidence that the content of TEK is also shifting. While experienced senior generations have a particular focus on ecological givens (e.g. weather conditions), younger generations are seeking ways that empower them to cope with transformations. This could be interpreted as herders no longer seeking short-term adaptation strategies such as long-distance movement called *Otor*, but also long-term strategies (e.g. improvement in livestock breeding). This could, in the worst case, lead to a loss of knowledge (e.g. about mobility and *Otor*). To counteract this, maintaining and archiving knowledge is a key aspect for keeping knowledge accessible if the societal situation shifts again and knowledge about plants, for example, becomes important (e.g. where the number of livestock decreases because of new breeds). From an ecological perspective, nomadism is an important component for a resilient steppe ecosystem. However, societal transformations towards urbanization point towards a contrary trend (Drees et al. 2022). For this, a societal discourse on future scenarios is needed and translated into regulations to find a resilient social–ecological state.

Regarding the intergenerational transfer of TEK, the qualitative interviews and focus group discussions confirm our second hypothesis that an intergenerational transfer within families, especially between parents and children, remains generally important. This result is in line with previous study results showing the significance of informal sources of ecological knowledge (Matias et al. 2018). However, additional knowledge sources within the family or community are gaining importance as part of the societal transformation, e.g. parents no longer working as herders. Thus, access to knowledge, and new knowledge sources is relevant when thinking about supporting sustainable development. The question is which level of political and institutional support might be best for supporting this transformation (e.g. central and local government; herder group level) (Jamsran 2010). Based on the data analysis, we claim that there is a need for local data, knowledge and face-to-face sharing. This is also a plea that has been made for years in the scientific community (Baival and Fernández-Giménez 2012; Fernández-Giménez et al. 2016). Here, herder organizations like PUGs, which according to our results currently have little effect on the application of TEK, might be an important institution that could take on the role of securing and passing on knowledge in the future.

From a methodological perspective, caution should be applied in interpreting the quantitative results, as it is not possible to infer causal relationships from correlations and the structural equation model alone. It is important to acknowledge that other confounding or unmeasured factors may contribute to the observed relationship between the gradient and ‘degree of TEK application’. Additionally, the absence of an established measurement for the ‘degree of TEK application’ in Mongolia poses a limitation. Thus, there is no comparative value of the ‘degree of TEK application’, which would allow for a temporal reference. For the first time, we have developed an operational approach for measuring the actual application of TEK. This process was based on the selection of seven questions informed by existing literature and previous in-depth interviews with herders in Central and Eastern Mongolia. Thus, this study represents a first attempt to quantify and standardize the measurement of TEK application in Mongolia, providing a valuable starting point for further investigation and the development of this construct.

## Conclusion

In international science-policy interfaces such as IPBES, the preservation and integration of local and traditional knowledge into land management have been emphasized for several years. However, it is also clear that the ecological and social conditions upon which TEK is based are changing dramatically, requiring adaptation to new social–ecological trajectories. The application of TEK empowers herders to cope with challenging conditions and also strengthens regional identity. This emphasizes with some urgency that knowledge must be applied flexibly, and that the pure ‘conservation’ of knowledge is not conducive. Thus, TEK has the potential to bridge the gap between tradition and modernity. New knowledge is needed from new sources and made by personal experience. The results of the study show that this is already a reality in Central and Eastern Mongolia. However, more studies are needed on how TEK and scientific knowledge complement each other, to identify and resolve conflicting perceptions of ecological conditions and dynamics, but also to find new ways (like social media) of sharing data and information. Stakeholders need to come together to consider the thematic content and nature of the transfer in order to perceive transformations. At the same time, Mongolian HHs must receive more support in passing on and sharing knowledge. The preservation and accessibility of knowledge, which currently has a minor significance in everyday life but could be important to the future, should play a key role in this.

## Supplementary Information

Below is the link to the electronic supplementary material.

Supplementary file1 (PDF 912 KB)
