# Supplementary material for: The role of traditional ecological knowledge, given the transformation of pastoralism in Central and Eastern Mongolia
Source: Ambio. 2024 Aug 29;53(12):1813–29. doi: 10.1007/s13280-024-02057-w (PMC11568112; doi:10.1007/s13280-024-02057-w)
Supplement: Supplementary file 1 — Supplementary file1 (PDF 912 KB) [file 13280_2024_2057_MOESM1_ESM.pdf]

The role of traditional ecological knowledge, given the transformation of pastoralism in Central and Eastern Mongolia

Sophie Peter[1,2], Sarah Nieß[1], Batbuyan Batjav[3], Nandintsetseg Dejid[2], Lukas Drees[1,2], Yun Jäschke[4], Ulan Kasymov[5], Sugar Damdindorj[6], Khishigdorj Dorjoo[7], Ganzorig Gonchigsumlaa[6], Denise Margaret S. Matias[8], Thomas Müller[2], Marion Mehring[1,2]

1. ISOE - Institute for Social-Ecological Research, Frankfurt am Main, Germany
2. Senckenberg Biodiversity and Climate Research Centre, Frankfurt am Main, Germany
3. CNPS - Centre for Nomadic Pastoralism Studies, Ulaanbaatar, Mongolia
4. Senckenberg Museum of Natural History Görlitz, Department of Botany, Germany
5. TUD - Dresden University of Technology
6. MULS - Mongolian University of Life Sciences, School of Economics and Business, Department of Agricultural and Applied Economics, Ulaanbaatar, Mongolia
7. Institute of Geography and Geo Ecology, Ulaanbaatar, Mongolia
8. HNEE - Eberswalde University for Sustainable Development

Appendices

Table S1. Relevant excerpt from the quantitative HH survey (2019, 2020, 2022)
*All answer options that contribute to the 'degree of TEK application' are marked with '(TEK)'

**(A) GENERAL INFORMATION**	
GPS Location	Latitude, longitude
**(B) HOUSEHOLD STRUCTURE**	
Gender	0=Male, 1=Female
Age	Years
Level of education	1=No schooling, 2=Attended primary school, 3=Attended secondary school, 4=Attended high school, 5=Attended college, 6=Attended university
Income in YEAR earned by HH member	MNT
How many years has the person been a herder?	Number of years
Since YEAR, has your household joined a herder organisation (Pasture User Group, herder group, cooperative, *nukhurlul* community, forest user group etc.)	Yes/No
**(D) LIVESTOCK INFORMATION**	
Total number of livestock by 31st of December, YEAR of the household<br>Type of livestock (goat, sheep, cattle/yak, horse, camel)	Total number (head)
**(E) PASTURELAND UTILIZATION AND HERDER MOBILITY**	
Respond according to seasonal mobility in YEAR (Note: these data are only for the family being surveyed).	Seasonal movement
Most important criteria for selecting pasture or camp	1=Vegetation condition **(TEK)***<br>2=Water source<br>3=Compliance with laws<br>4=Presence of gazelles **(TEK)**

	5=Weather conditions **(TEK)**<br>6=Staying together with family **(TEK)**<br>7=Both vegetation and water source **(TEK)**<br>8=Other (………………) (Responses were categorized as **TEK** or not)
**(G) DISASTER**	
How do you predict that a *dzud* will occur? (Select as many answers as possible)	Monkey year **(TEK)**<br>Look of the moon, sun and stars (e.g., 2nd day of every month according to the lunar calendar) **(TEK)**<br>Large migration of gazelle **(TEK)**<br>In autumn: high abundance of Stipa **(TEK)**<br>Drought in summer **(TEK)**<br>Others (specify ....) (Responses were categorized as **TEK** or not)<br>No, we cannot predict
How do you predict that a drought will occur? (Select as many answers as possible)	Lack of rain in summer **(TEK)**<br>Shortage of water **(TEK)**<br>Less rain and snow in spring **(TEK)**<br>Reduction of biomass **(TEK)**<br>Late rain in spring **(TEK)**<br>Too much heat in spring and summer **(TEK)**<br>Too much wind in spring and summer **(TEK)**<br>Others (specify........................) (Responses were categorized as **TEK** or not)<br>No, we cannot predict.
Where did you learn to predict that a *dzud* or drought will occur?	Parents<br>Other relatives<br>Friends<br>School<br>Self-taught<br>Training by governmental organisation or NGO<br>Other (specify……………………)
What do you do for herding your livestock during a drought or *dzud*?	Move long distances (Otor) **(TEK)**<br>Prepare own hay **(TEK)**<br>Buy hay<br>Reserve pasture<br>Change summer camps frequently **(TEK)**<br>Get help from relatives or friends<br>Get support from local government<br>Other (specify……………………) (Responses were categorized as **TEK** or not)
In your opinion, has there been any change in extreme weather events since you came here (drought, *dzud*)?	Frequency increased<br>Became more intense or harsh<br>Lasts longer<br>Other

**(M) LIVESTOCK LOSS**	
If the animals are sick with disease, how do you treat them?	Go to the veterinary<br>Use traditional remedies **(TEK)**<br>No measures taken<br>Other
If the animals are injured (e.g. a broken leg) how do you treat them?	Go to the veterinary<br>Use traditional remedies **(TEK)**<br>No measures taken<br>Other
If you use traditional remedies, what are these? (Enumerate)	For animal sickness:<br>For animal injuries:
Where did you learn to use traditional remedies for curing animal disease or injuries?	Parents<br>Other relatives<br>Friends<br>School<br>Self-taught<br>Training by governmental organization or NGO<br>Other
**(Q) NEW QUESTIONS FOR 2020**	
From your experience what are the most important factors to judge if the pasture is good. Please rank according to importance for the following 4 factors.	Total vegetation cover<br>Vegetation height<br>Aboveground biomass<br>Forage quality (existence of special desirable plants for livestock) **(TEK: Rank 1 or 2)**<br>Other
How do you tell if pasture conditions are becoming worse? Please select the most important factors that apply (multiple answers)	Area of bare/sandy soils increases<br>Grass production/plant height or size decreases<br>Number of plant species decreases **(TEK)**<br>Number of forage plants decreases **(TEK)**<br>Quality of meat production decreases (meat does not taste as good any more)<br>Quantity of meat production decreases<br>Quality of milk products decreases<br>Quantity of milk products decreases<br>Number of Brandt's vole increases **(TEK)**<br>Yellowing of the colour of the grasses in summer **(TEK)**<br>Other (Responses were categorized as **TEK** or not)

Table S2. Detailed quantitative results of the seven questions selected for the HH survey to address the 'degree of TEK application'

Extreme events (*dzud* and drought)	*Dzud* is an extreme weather event in Mongolia that leads to extreme climatic conditions (e.g., winter storm). Forecasting such a weather event is essential for herders to save livestock. Therefore, we asked the following question: "How do you predict that a *dzud* will occur?" (Q. 83). Most HH answered that they predict a *dzud* based on summer drought (30.45%), followed by 'In autumn: high abundance of Stipa' (11.42%) and 'Gazelle migration' (11.07%). Other methods not directly surveyed (e.g. heavy first snowfall) were also listed by some HH. However, 20.76 % also indicated that they could not predict a *dzud*. Most HH purchase hay to deal with a *dzud* (83.84%), followed by moving long distances (Otor) (57.09%), which is the traditional way of coping with *dzud*.<br><br>In terms of predicting drought conditions, the most frequently mentioned answer was 'lack of rain in summer' (57.44%). This was followed by 'less rain & snow in spring' (38.75%) and 'late rain' (30.1%). Overall, only 18.68% of HH indicated that they could not predict a drought. To deal with the drought, most HH reported going on Otor (83.84%) or changing camp location more frequently (30.89%).
Livestock health	One more important work field for herding is maintaining the health of livestock. Overall, 76.12% of HH reported that they use traditional remedies when their livestock is sick, while 60.91% go to the veterinarian and use traditional remedies. The most commonly mentioned traditional remedies in this case are syringe (17.65%), remedies and syringe (11.07%), and pricking (9.34%). HH are even more aligned when it comes to treating injured animals. 93.08% use traditional remedies in these situations, while only 32.18% go to the veterinarian and use traditional remedies. Remedies used are 'wrap the legs' (72.3%) and 'wrap the wound' (17.65%).
Pasture quality	The health of the livestock is also strongly related to the selection of high-quality pasture. The most important criterion for selecting a pasture or camp was 'vegetation & water source' (51.39% - 58.48%) for all seasons. Herders were also asked to rank the most important factors for identifying good pastures. Overall, 62.98% of HH selected "forage quality" as the most or second most important factor, which is in line with TEK. Next, households were asked how they identified a degradation of pasture conditions. Multiple answers were possible. It is interesting to note that most HH (85.12%) mentioned one or more

	methods that we consider to be TEK: number of plant species decreases, number of forage plants decreases, number of Brandt's vole increase and yellowing of the color of the grasses in summer. In addition, 'reduction in grass production/plant height or size' was mentioned very frequently (84.48%).

Category	TEK Status Item	Range	Sample Range	Sample Mean	Sample Median	Sample SD
Extreme events	Predict dzud	0-7	0-5	2.00	2.00	1.19
	Predict drought	0-9	0-8	3.14	3.00	1.93
	Adapt drought	0-4	0-3	1.40	1.00	0.67
	Adapt dzud	0-4	0-4	1.16	1.00	0.77
Livestock health	Livestock health	0-2	0-2	1.70	2.00	0.54
Pasture quality	Pasture selection	0-4	0-4	3.60	4.00	0.99
	Pasture degradation	0-6	0-6	2.50	2.00	1.24
TEK appl. degree		0-36	3-27	15. 50	16.00	4.14

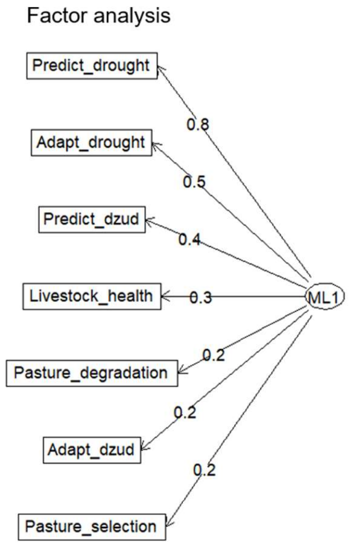

Figure S1. Factor analysis 'degree of TEK application'

Table S3. Interview guideline for the qualitative interviews and focus group discussions (2019, 2020, 2022)

YEAR(S)	TOPIC	Answer categories
**2019, 2020, 2022**	**LOCATION**	
	Location	*Aimag*<br>*Soum*<br>*Bag*
**2019, 2020, 2022**	**DEMOGRAPHIC**	
	How many people are there in your family living with you?	Gender<br>Age<br>Education

**2020**	**SEASONAL MOVEMENT (2020)**	
	Can you provide your knowledge on how you choose seasonal pastures? Based on what criteria do you make decisions on pasture use and mobility?	Source of knowledge, where does this knowledge come from
	What has changed in the last 10 years? Seasonal grazing area? Distance, number of movements?	Open question
	Tell us about the risks and hazards (drought, *dzud*, dust storm, fire, etc.) that you face as pastoralists. What measures were taken in that year? In times of natural disaster which of the traditional knowledge helped?	Open question
	**ORGANIZATION/ HERDER GROUP**	
2019	Do you belong to any formal herder group?	Herder group<br>Pasture user group<br>Cooperative<br>*Nokhorlol*<br>Others
2019	Based on what information do you make decisions on pasture use and mobility? (List all that are relevant in order of importance, e.g. pasture condition vegetation cover, plats, etc.)	Open question
2019	Tell us about the risks and hazards that you face as pastoralists. Which have been the most challenging and why?	Open question
2019	Do you rely only on previous experience or are you also guided by information/knowledge from friends/relatives etc?	Open question
2019	Which of the following measures did you take over the last 12 months (circle all that apply):	Fed animals stored hay<br>Fed animals purchased fodder concentrate<br>Took animals on Otor<br>Grazed animals on reserve winter pasture<br>Other
2019	How have members of your community coped with environmental change and other challenges?	Open question
2019	In your community last summer, were the pasture conditions	Open question

	(forage quality and quantity) better than usual, about the same as usual, or worse than usual?	
2019	Do you want to make any changes in your pastoral management practices in the next 5 years? If so, what changes do you plan to make?	Open question
2020	Do you belong to any herder group? Any formal herder group? Can you provide information about your group? Which of the following list of activities do members of the group engage in together? For example : monitor pasture conditions; collectively set aside and protect *dzud* reserve pastures; determine how many livestock can graze in certain pastures; manage the use of wells together; repair or maintain a well or wells together	Open question
2020	How have members of your community coped with environmental change and challenges in the past? Can you assess the level of trust in your community?	Open question
**2020**	**KNOWLEDGE**	
	How do you build your knowledge and experience? Who do you share your knowledge and experience with? Do you rely only on previous experience or are you also guided by information from friends/relatives etc?	Open question
	Have you exchanged experiences with officials on challenges that you or your community face. Have you attended any training on traditional knowledge?	Open question
	In your opinion, what is the most important traditional knowledge on mobility?	Open question
**2020**	**NATURAL CONDITIONS**	
	How has the current condition of summer pastures in your *soum* changed in the last 5 years?	Open question
	In the last 5 years, have you had any land, pasture, or water disputes?	Open question
